# Novel Metabolic Signatures of Prostate Cancer Revealed by ^1^H-NMR Metabolomics of Urine

**DOI:** 10.3390/diagnostics11020149

**Published:** 2021-01-20

**Authors:** Bo Yang, Chuan Zhang, Sheng Cheng, Gonghui Li, Jan Griebel, Jochen Neuhaus

**Affiliations:** 1 Department of Urology, University of Leipzig, 04103 Leipzig, Germany; paulyang228@hotmail.com (B.Y.); Chuan.Zhang@medizin.uni-leipzig.de (C.Z.); 2 Department of Urology, Zhoupu Hospital, Shanghai University of Medicine & Health Sciences, Shanghai 201318, China; 3 Department of Urology, Sir Run Run Shaw Hospital, Zhejiang University School of Medicine, Hangzhou 310016, China; 3193119@zju.edu.cn; 4 Leibniz Institute of Surface Engineering (IOM), Permoserstraße 15, 04318 Leipzig, Germany; jan.griebel@iom-leipzig.de

**Keywords:** prostate cancer, urine metabolomics, ^1^H-Nuclear Magnetic Resonance, metabolite biomarkers

## Abstract

Prostate cancer (PC) is one of the most common male cancers worldwide. Until now, there is no consensus about using urinary metabolomic profiling as novel biomarkers to identify PC. In this study, urine samples from 50 PC patients and 50 non-cancerous individuals (control group) were collected. Based on ^1^H nuclear magnetic resonance (^1^H-NMR) analysis, 20 metabolites were identified. Subsequently, principal component analysis (PCA), partial least squares-differential analysis (PLS-DA) and ortho-PLS-DA (OPLS-DA) were applied to find metabolites to distinguish PC from the control group. Furthermore, Wilcoxon test was used to find significant differences between the two groups in metabolite urine levels. Guanidinoacetate, phenylacetylglycine, and glycine were significantly increased in PC, while L-lactate and L-alanine were significantly decreased. The receiver operating characteristics (ROC) analysis revealed that the combination of guanidinoacetate, phenylacetylglycine, and glycine was able to accurately differentiate 77% of the PC patients with sensitivity = 80% and a specificity = 64%. In addition, those three metabolites showed significant differences in patients stratified for Gleason score 6 and Gleason score ≥7, indicating potential use to detect significant prostate cancer. Pathway enrichment analysis using the KEGG (Kyoto Encyclopedia of Genes and Genomes) and the SMPDB (The Small Molecule Pathway Database) revealed potential involvement of KEGG “Glycine, Serine, and Threonine metabolism” in PC. The present study highlights that guanidinoacetate, phenylacetylglycine, and glycine are potential candidate biomarkers of PC. To the best knowledge of the authors, this is the first study identifying guanidinoacetate, and phenylacetylglycine as potential novel biomarkers in PC.

## 1. Introduction

Prostate cancer is one of the most commonly cancers and the leading cause of cancer-related deaths in men worldwide [1]. Serum prostate specific antigen (PSA) level and digital rectal examination (DRE) constitute the major screening tests for prostate cancer (PC) diagnosis, while the transrectal ultrasound-guided prostate biopsy provides the final confirmation of cancer presence [2]. PSA level has been extensively used as a biomarker to detect PC. Nevertheless, due to prostate physiology, PSA testing results in a large frequency of false positives leading to numerous men each year undergoing unnecessary prostate biopsy procedures [3,4,5,6,7]. Hence, a non-invasive, cost-effective, efficient, and reasonably accurate test for early identification of PC is urgently needed.

Compared with serum, urine is easier to obtain and handle, needs less sample preparation, and has higher amounts of metabolites and lower protein content [8,9,10]. Therefore, in attempt to solve this diagnostic dilemma, many previous studies have focused on urinary metabolomic profile, to identify the predictive biomarkers for PC [11,12,13,14]. However, to date, no single urine biomarker/biomarker panel meets the requirements for highly sensitive, and specific detection of PC. Therefore, biomarker discovery in relation to PC continues to be an active area of research.

Nuclear magnetic resonance (NMR) spectroscopy is a powerful analytical approach for both identification and quantification of analytes with superior advantages, such as good reproducibility and simple sample processing. In the last decade, NMR has been applied toward identifying metabolic alterations in PC that may provide clinically useful biomarkers [15,16,17,18,19]. ^1^H-NMR spectroscopy followed by multivariate analysis is a systems biological approach that has been used to identify essential changes in metabolism. Therefore, metabolomics profiling offers a robust methodology for understanding the biochemical process of diseases.

**Figure 1:**
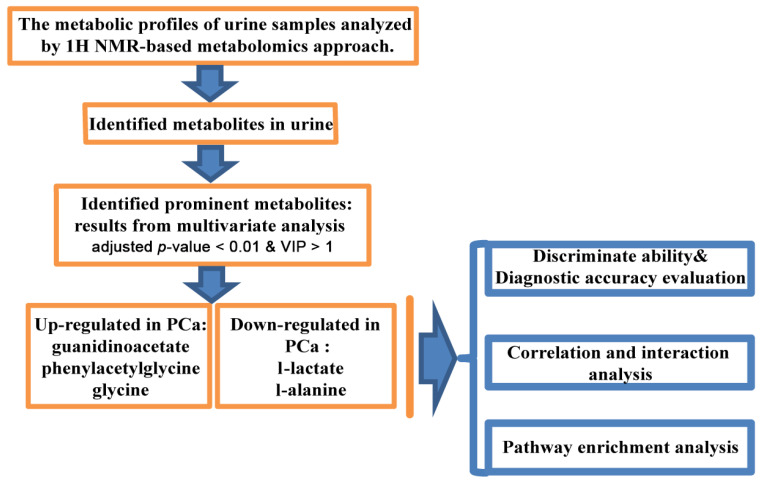
Study design. The workflow of the analysis steps.

Our current study aimed to identify novel biomarkers in the urine and to investigate the possible function and role of potential biomarkers in PC. Based on ^1^H-NMR, we identified 20 metabolites from urine samples. All spectra were analyzed by multivariate statistical analysis to extract the vital variables. Moreover, to evaluate the discrimination ability of the variables for diagnosis of PC. Additionally, metabolomics analysis cannot provide direct information about the active pathways related to the diseases. Furthermore, the regulation of the reactions and metabolic programs still need to be addressed [20]. Figure 1 summarizes the study design and workflow.

## 2. Materials and Methods

### 2.1. Clinical Samples Selection and Ethics Statement

Urine samples were collected from PC patients from January 2017 to December 2018 from Sir Run Run Shaw Hospital, HangZhou and Zhoupu Hospital, Shanghai, China. Clinical diagnosis of individuals was performed according to serum PSA, DRE, biopsy results/pathological results after operation and Gleason score. A total of 50 patients with prostate cancer were included in this study. The control group consisted of 50 non-cancerous men, who were without evidence of PC, based on PSA levels, negative findings in imagological examination and DRE. Clinical and demographics characteristics of the individuals are shown in Table 1.

Patients recruitment and sampling procedures were performed in accordance with the Declaration of Helsinki and applicable local regulatory requirements and laws. All patients provided written informed consent. Ethical approvals were obtained from the local ethics committees of the Sir Run Run Shaw Hospital affiliated to Zhejiang University (Ethical review approval number: 20190725-290) and Shanghai University of Medicine & Health Sciences (Ethical review approval number: HMMEP-2016-017).

### 2.2. Sample Preparation and ^1^H-NMR Based Metabolomics Analysis

Midstream urine samples of all PC patients and controls were taken in the morning during standard clinical routine procedure. The samples were frozen within 1 h after collection and stored at −80 °C. At the time of ^1^H-NMR analysis, urine samples were thawed in an ice-water bath. Where not otherwise stated, chemicals were from Sigma-Aldrich Trading Co., Ltd., Shanghai, China. Two hundred µL of phosphate buffered saline (PBS) solution (0.1ml Na_2_HPO_4_ and 0.1ml NaH_2_PO_4_; 10% D_2_O and 0.03% TSP (trimethylsilylpropionic acid-d_4_ sodium salt; pH 7.4) was added and the samples were centrifuged at 13,000 rpm for 20 min. After this, 550 µL of the supernatants were transferred to a 5-mm NMR tube for analysis. ^1^H-NMR spectral acquisition was performed using a Bruker Avance III NMR spectrometer equipped with 600 MHz magnets Ultrashield Plus (spectrometer frequency: 600.13 MHz; Bruker BioSpin Corporation, Billerica, MA. USA). All ^1^H-NMR experiments were performed at 25 °C.

All spectra were phase and baseline corrected, and chemical shifts were adjusted with reference to TSP signal using MestRenova 6.2 software (Mestrelab Research S.L., Santiago de Compostela, Spain). The spectra were binned into 0.02 ppm buckets between 0.52 and 9.30 ppm, and the region between δ 4.32 and 6.10 ppm, including the water (δ 4.32 and 5.26 ppm), and urea signal (δ 5.58 and 6.10 ppm) regions, was excluded from the analysis to avoid interference arising from differences in water suppression and variability from the urea signal.

### 2.3. Data Modelling and Statistical Analysis

**Figure 2:**
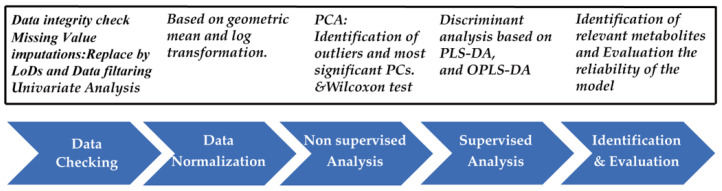
General scheme of the data modelling and statistical analysis procedures (sample size, *n* = 100; variables size, *n* = 20).

Before data analysis, we checked the data integrity. All missing values, zeros, and negative values were replaced by the 1/5 of the minimum positive value of each variable [21,22]. In addition, after the replacement, we compared the two data sets: before replacement and after replacement. We made sure that all the necessary information has been collected, and that there was no significant difference between the two data sets (Table S1) and subgroups (cancer group and control group) (Tables S2 and S3). The normalization of the spectra was performed by R statistical package 4.0.2 (http://www.r-project.org) based on geometric mean, and generalized log transformation was performed to make features more comparable (Figure S1 Supplementary Materials; Figure 2).

### 2.4. Identification of Relevant Metabolites

For identification of relevant metabolites, we used several statistical approaches resulting in the definition of a subset of metabolites identified by at least two methods. The Multivariate statistical analysis was carried out using R packages “MetaboAnalyst” [21,22,23],” ropls” [24], ”mixOmics” [25]. Principal component analysis (PCA) as a non-supervised statistical method, we used to uncover the outliers and the directions that best explain the variance in the dataset. Partial Least Squares discriminant analysis (PLS-DA), and Orthogonal Partial Least Squares discriminant analysis (OPLS-DA) were used to reduce the number of metabolites in high-dimensional data to produce robust and easy-to-interpret models, and to identify spectral features that drive group separation. Subsequently, based on R, Wilcoxon rank sum test was performed to find the difference between the cancer group and control group. The difference was considered significant at a Bonferroni-adjusted *p*-value < 0.05.

The variable importance in projection (VIP), and corresponding loading/contribution value in each model was used to identify the variables responsible for distinguishing. Furthermore, a permutation test with 100 permutations was employed to validate the performance of PLS-DA models and OPLS-DA models. For quality criterion we chose in PCA model, R^2^X > 0.4; in PLS-DA or OPLS-DA, R^2^Y (goodness of fit parameter) and Q^2^ (predictive ability parameter) > 0.5 [26,27].

### 2.5. Acquisition of the Pathways and Biological Processes Corresponding to Metabolites

To explore the significance of a specific metabolite for prostate cancer, we used public databases to identify associated pathways. We focused on the most prominent metabolites defined by several criteria: (i) the metabolite was at least recommended in two different models (PCA, PLS-DA, or OPLS-DA); (ii) Wilcoxon test adjusted *p*-value < 0.01; (iii) VIP-values of the OPLS-DA >1.

Furthermore, the R package “MetaboAnalyst” [21,22,23] was performed analyze the contribution of the metabolites in depth. To implement a knowledge-based network of metabolite-metabolite interactions we used the Search Tool for Interactions of Chemicals (STITCH) database [28]. We also performed a Metabolite Sets Enrichment Analysis (MSEA), including pathway enrichment analysis based on the Kyoto Encyclopedia of Genes and Genomes (KEGG) and the Small Molecule Pathway Database (SMPDB) [29,30]. A hypergeometric test was used to evaluate whether a particular metabolite set is represented, and the metabolite set contains at least more than 2 metabolites in the given compound list. Additionally, one-tailed *p*-values were provided after adjusting for multiple testing. A *p*-value < 0.05 was considered statistically significant.

### 2.6. Statistics

All statistical analyses were performed using SPSS software (version 26; IBM Corp., Armonk, NY, USA) or R statistical package 4.0.2 (http://www.r-project.org). Univariate analysis was performed using ANOVA, *t*-test, Wilcoxon test, hypergeometric test and permutation test. Bonferroni was used to adjust *p*-values. The correlation analyses were performed by Pearson’s test. Multivariate analyses were also performed using the PCA, PLS-DA, and OPLS-DA model. Subsequently, we used binary regression and a linear fitting model to do receiver operating characteristic (ROC) curve analysis to evaluate the performance of the metabolite or metabolite panel for the prediction of PC. *p*-values < 0.05 or adjusted *p*-values < 0.05 were considered statistically significant.

## 3. Results

### 3.1. Metabolites in Urine Samples of PC

**Figure 3:**
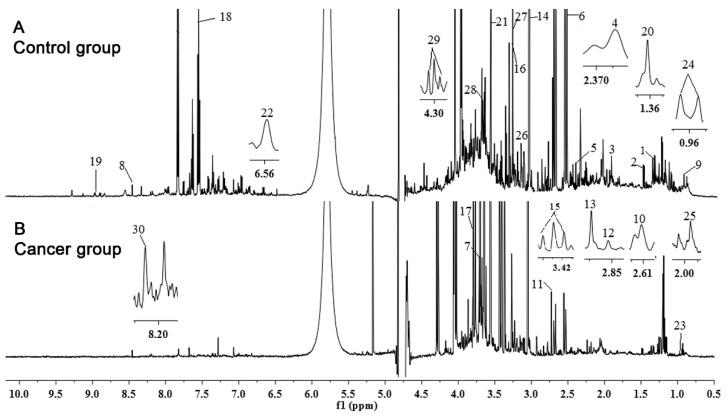
Representative 600 MHz ^1^H-NMR spectrum and assignment of identified metabolites (digits from 1 to 30) in two representative urine samples. Signals were analyzed from δ 0.52 to 9.30 ppm, excluding water and urea regions (δ 4.32–6.10 ppm). (**A**) Control group; (**B**) Cancer group; f1 (ppm) = chemical shift to TSP.

NMR offers the opportunity of quantifying metabolites directly from ^1^H-NMR metabolite profiles through analyzing the chemical shift, coupling constant, and shapes of peaks from NMR experiments, and to identify the metabolites based on existing public databases and literature reports [31,32,33,34,35,36]. Typical ^1^H-NMR spectra were derived from urine samples of the PC group and the Control group; interesting metabolites were identified (labeled as digits from 1 to 30 in Figure 3).

The region at 0.0–3.10 ppm shows aliphatic compounds including prominent signals from organic acids and amino acids, such as L-alanine, citric acid, pyruvate, succinate, and L-lactate; the region at 5.5–9.0 ppm shows aromatic compounds, such as hippurate and also formate, deeply downshifted due to the adjacent carboxy group. Additionally, moieties and chemical shifts of the 30 metabolites were summarized in Table S4. Finally, after removal of metabolites with overlapping signals, we got 20 metabolites which were further analyzed in this study (Table 2). For intensity quantification, the peak areas of these 20 metabolites were integrated using sodium trimethylsilyl propionate (TSP) as standard for further analysis.

### 3.2. Identification of Important Metabolites and the Metabolic Changes

**Figure 4:**
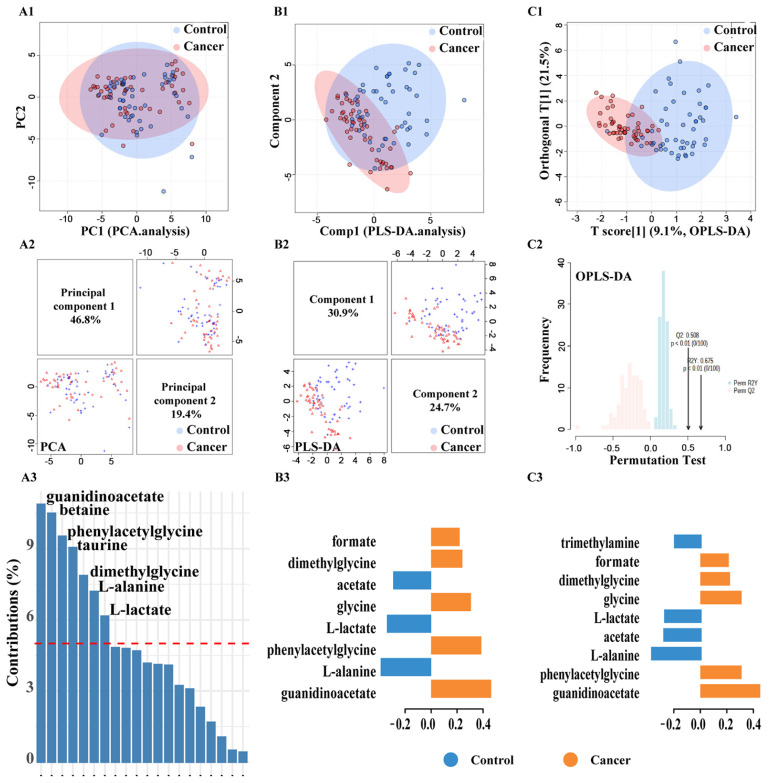
Metabolic pattern recognition analysis. Classifying PC from non-cancerous men based on the metabolomic profiles in the urine; (**A1**) PCA based on the first two principal components; (**A2**) sample scatterplot displays the first two components in each data set in PCA; (**A3**) contribution of each feature selected on the first component in PCA; (**B1**) PLS-DA based on the first two components; (**B2**) sample scatterplot display the first two components in each data set in PLS-DA; (**B3**) loading plot weights of each feature selected on the first component of PLS-DA; (**C1**) OPLS-DA based separation of the groups; (**C2**) internal validation of the corresponding OPLS-DA model by permutation analysis (n = 100); fraction of the variance of descriptor class response (Y) (R2Y) = 0.675 (Green bar), *p*-value < 0.01; fraction of the variance predicted (cross-validated)(Q2) = 0.508 (Red bar), *p*-value < 0.01; (**C3**) loading plot weights of each feature selected from OPLS-DA; The color in B3 and C3 indicates the class in which the variable has the maximum level of expression; control = blue; cancer = orange.

PCA, PLS-DA, and OPLS-DA were performed to evaluate the metabolic pattern changes in PC patients compared to non-cancerous controls. PCA could not distinguish the cancer patients from the non-cancerous cases (Figure 4(A1)). The first two principal components (PC) explained 66.2% variables; however, no trends in differences were detected (Figure 4(A2)). Based on the contribution value, we obtained the top seven metabolites, including guanidinoacetate, betaine, phenylacetylglycine, taurine, dimethylglycine, L-alanine, and L-lactate (Figure 4(A3)) (Table S3). The goodness of fit of the PCA model was R^2^X = 0.607.

Key numbers are related to the metabolite numbering in Figure 1; the variable importance in the projection (VIP) values were obtained from the OPLS-DA model.

If PLS-DA was used as classification model, we found a trend to distinguish cancer from the control (Figure 4(B1)). In this model, the first two principal components explained 55.6% of the variance (Figure 4(B2)). Based on the |loading values| > 0.2, we found 8 significant metabolites: guanidinoacetate, L-alanine, phenylacetylglycine, L-lactate, glycine, acetate, dimethylglycine, and formate (Figure 4(B3)) (Table S3). Furthermore, the PLS-DA performance was assessed by the goodness of fit R^2^Y = 0.628 and quality assessment statistic Q^2^Y = 0.447; the outcome indicated good class separation and a moderate predictive ability.

Further improvement in discrimination of the sample groups was achieved by using the OPLS-DA model (Figure 4(C1)). Based on the |loading values| > 0.2, OPLS-DA identified nine critical metabolites: guanidinoacetate, L-alanine, phenylacetylglycine, acetate, l-lactate, glycine, dimethylglycine, formate, and trimethylamine (Figure 4(C3)) (Table S3).

As shown in Figure 4, we could not completely discriminate the two groups based on PLS-DA and OPLS-DA scores plot. However, more samples were separated in OPLS-DA in contrast to the PCA method. This OPLS-DA model showed a proper fitting of the data (R2Y = 0.675, *p*-value < 0.01), and exhibit predictive power (Q2 = 0.508, *p*-value < 0.01) (Figure 4(C2)).

The variable importance in the projection (VIP) values of all peaks from OPLS-DA models were taken for selection, and those variables with VIP > 1 [37] were considered as potential biomarker candidates for group discrimination (Table 2). Accordingly, metabolomics revealed prominent alterations in seven metabolites: guanidinoacetate, l-alanine, phenylacetylglycine, acetate, l-lactate, glycine, and dimethylglycine (Table S3). In summary, the ^1^H-NMR spectra potentially discriminate the urine samples between PC patients and controls.

**Figure 5:**
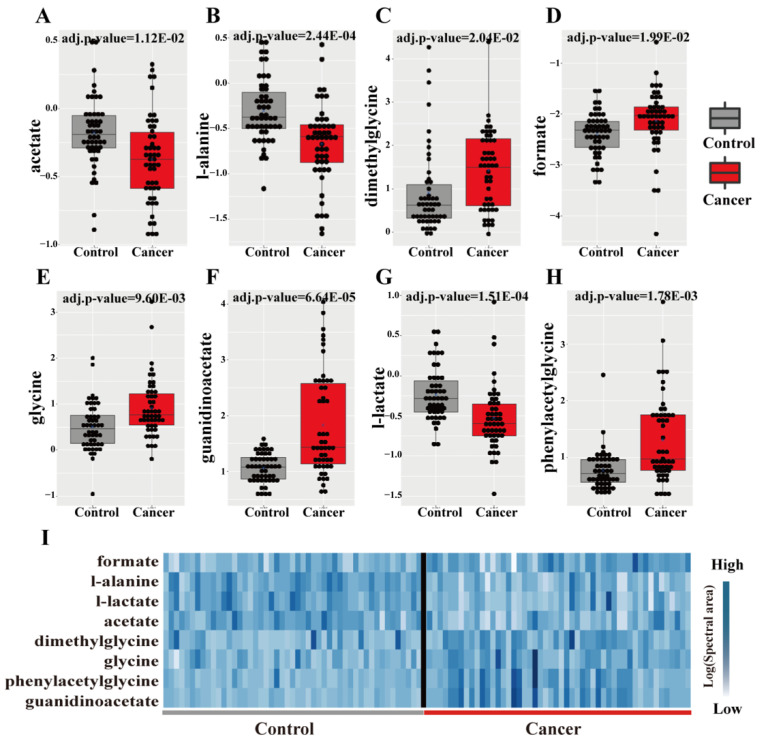
Wilcoxon test results and hierarchical clustering of the metabolites. (**A**–**H**) Box plots of levels of significant metabolites based on Wilcoxon test; (**I**) hierarchical clustering of the significant metabolites; the samples on the left of the black bar are non-cancerous samples (control group, *n* = 50); the samples on the right of the black bar are PC samples (*n* = 50); values in the heatmap = Log (Spectral area); *p*-values were Bonferroni-adjusted.

For direct comparison of the levels of the 20 metabolites, an integrated strategy combining Wilcoxon analysis was used to identify critical metabolites between the PC and the control group. We compared the urinary metabolomic profiles of the two groups, based on the Bonferroni method of *p*-value adjustment. The analysis revealed a total of eight significant metabolites (adjusted *p*-value < 0.05): guanidinoacetate, l-lactate, l-alanine, phenylacetylglycine, glycine, acetate, formate, and dimethylglycine (Figure 5A–I).

### 3.3. Acquisition of the Most Prominent Metabolites, Correlation Analysis, and ROC Analysis

**Figure 6:**
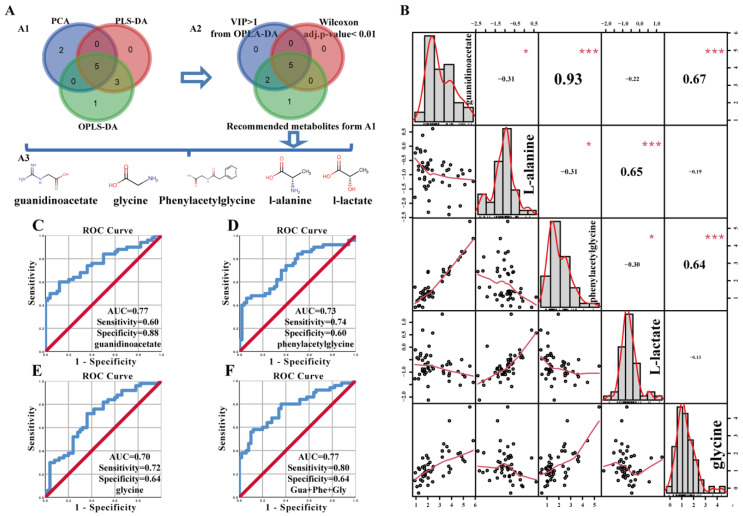
Correlations between the five most prominent metabolites and the representative ROC analyses. (**A**) Venn diagram describing the overlapping results from different models. (**A1**) overlapping of the significant metabolites from different models, revealed 8 metabolites at least recommended in two models. Significant metabolites detected by PCA (7 metabolites, marked in blue), PLS-DA (8 metabolites, marked in red), and OPLS-DA (9 metabolites, marked in green); (**A2**) overlapping of the significant metabolites from three different models revealed 5 metabolites, which were common between OPLS-DA (blue circle, VIP > 1, *n* = 7), metabolites with Wilcoxon test adjusted *p*-value < 0.01 (red circle, n = 5), and overlap result was obtained from A1 (green circle, *n* = 8); (**A3**) identification and chemical formula of the 5 significant metabolites. (**B**) Correlation between the 5 metabolites in PC: (i) the histogram of the kernel density estimation and distribution of each variable is shown on the diagonal, (ii) On the bottom of the diagonal: the bivariate scatter plots with a fitted line are displayed, (iii) On the top of the diagonal: the value of the correlation plus the significance level as stars; each significance level is associated to a symbol: *p*-values (0, 0.001, 0.01, 0.05, 1) relate to symbols (“***”, “***”, “**”, “*”, “ ”); the number in the charts is the Pearson’s correlation coefficient (*r*); (iv) Numbers at the sides of the charts indicate the range of variable values are depicted as Log(Spectral area). (**C**–**F**) Representative ROC curves showing the diagnostic accuracy (AUC) based on guanidinoacetate (**C**), phenylacetylglycine (**D**), glycine (**E**), and in (**F**) the combination of the three metabolites: guanidinoacetate (Gua), phenylacetylglycine (Phe), and glycine (Gly).

Regarding the criterion of the most prominent metabolites: (i) the metabolite was at least recommended in two different models, from PCA, PLS-DA and OPLS-DA (Figure 6 (A1)) (Table S5); (ii) Wilcoxon test adjusted *ps* < 0.01 (Figure 6 (A2); Table S5); (iii) the VIP-values of the OPLS-DA >1 (Figure 6 (A2); Table S5). Herein, after the overlapping progression, we focused on the five most prominent metabolites: guanidinoacetate, phenylacetylglycine, glycine, L-lactate and L-alanine (Figure 6 (A3); Table S5)**.** Interestingly, based on the Human Metabolome Database (HMDB) [37], guanidinoacetate and phenylacetylglycine have not been detected in prostate tissue, so far (Table S4). We found a strong positive correlation between guanidinoacetate and phenylacetylglycine (Pearson’s correlation coefficient; *r* = 0.93, *p*-value < 0.001), and moderate positive correlations between l-alanine and l-lactate (*r* = 0.65, *p*-value < 0.001), guanidinoacetate and glycine (*r* = 0.67, *p*-value<0.01), and phenylacetylglycine and glycine (*r* = 0.64, *p*-value < 0.001) (Figure 6B).

ROC analysis of significant metabolites in multiple t-test revealed for guanidinoacetate an AUC of 0.77 (sensitivity = 60%, specificity = 88%; Figure 6C), phenylacetylglycine an AUC of 0.73 (sensitivity = 74%, specificity = 60%; Figure 6D), and glycine an AUC of 0.70 (sensitivity = 72%, specificity = 64%; Figure 6E). The AUCs of l-alanine and l-lactate were lower than 0.70, respectively (data not shown).

Based on a linear fitting model, various combinations were evaluated for their ability to predict PC. The combination of guanidinoacetate, phenylacetylglycine and glycine identified PC with an AUC = 0.77, sensitivity = 80%, and specificity = 64%. However, while improving the sensitivity from 60% to 80% (*p*-value = 0.03), this combination did not significantly improve the diagnostic probability of PC (Figure 6F). The combination of guanidinoacetate, phenylacetylglycine, glycine, l-alanine, and l-lactate showed less performance (AUC = 0.65, sensitivity = 52%, specificity = 80%; data not shown), as did the combinations of l-alanine and l-lactate and others (AUCs < 0.7 with low specificity and sensitivity; data not shown).

### 3.4. Subgroup Analysis

To explore the property of the metabolites to separate between different PC stages, we compared the urine levels of the five metabolites L-lactate, L-alanine, glycine, guanidinoacetate, and phenylacetylglycine in different subgroups of PC. Three metabolites: glycine, guanidinoacetate, and phenylacetylglycine showed significant differences between low GS ≤ 6 and high GS ≥ 7 when using the biopsy GS (GS (pre)) or final post-surgery GS (GS (post)) for stratification (Figures S3 and S4, ANOVA with Bonferroni-adjusted *p*-values, *p* < 0.05).

In addition, we found significant differences in the urine levels of glycine, guanidinoacetate and phenylacetylglycine between PSA-groups (low PSA: ≤10 ng/mL and high PSA: >20 ng/mL), while l-lactate and l-alanine were not different (Figure S5). Comparison of TNM or risk groups did not reveal significant differences (data not shown).

### 3.5. Analysis of the Metabolite Interaction Networks and Corresponding Pathways

**Figure 7:**
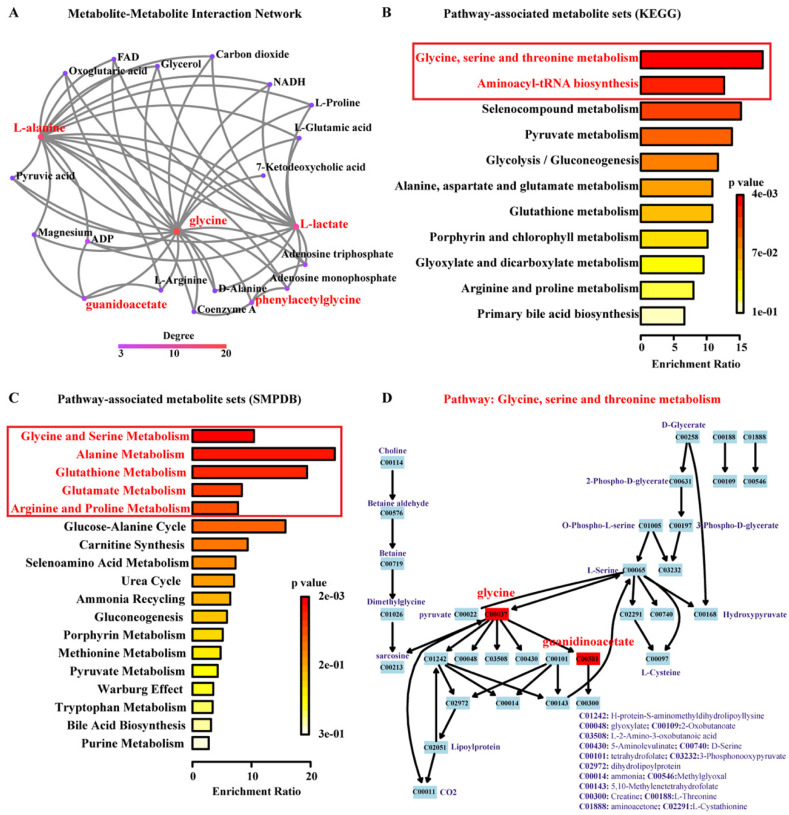
Interaction network analysis and pathways associated with the five identified metabolites. (**A**) Interaction map of the annotated metabolites; nodes are color coded for the degree of the metabolite interactions; (**B**) pathways associated with the most prominent metabolites based on KEGG analysis; (**C**) pathways associated with the most prominent metabolites based on SMPDB analysis; (**D**) detailed view of the “Glycine, serine and threonine metabolism” (KEGG map00260) as the most significant pathway according to the KEGG analysis; the numbers in the boxes represent the IDs of annotated metabolites in KEGG database; prominent metabolites as a result of the current analysis are marked in red. Key signaling pathways with *p*-values < 0.05 were marked in boxes with red font in (**B**,**C**).

The network explorer module is a comprehensive tool to describe potential impacts, and to visualize interactions between metabolites. Network analysis highlights potential functional relationships between a broad set of annotated metabolites. Based on the degree of interaction cut-off value >2, we found another 16 annotated metabolites potentially interacted with the five metabolites defined above, and we also found 53 different interactions among them (Figure 7A).

According to the *p*-values from the pathway enrichment analysis, the pathways containing at least two components of the five prominent metabolites are listed in Figure 7B. Based on KEGG database analysis, “Glycine, serine, and threonine metabolism” and ”Aminoacyl-tRNA biosynthesis” were the associated pathways with *p*-value < 0.05. Figure 7C describes the five associated pathways based on SMPDB, such as “Glycine and Serine Metabolism” and “Arginine and Proline Metabolism”. Figure 7D Detailed view of the “Glycine, serine, and threonine metabolism” as the most significant pathway.

## 4. Discussion

### 4.1. The Location and Expression of Metabolites in PC

Notably, urine is a challenging bio-specimen used for biomarker discovery due to its compositional variability [38,39]. Multiple factors can affect the composition and quality of urine liquid biopsy, such as disease state, prescription taken by individuals, diet, gender, and collection time [38,39]. In the present study, multivariate statistical models were used to identify reliable candidate biomarkers of PC. Eventually, we found that guanidinoacetate, phenylacetylglycine, glycine, l-lactate, and l-alanine were the most prominent metabolites.

Lima and colleagues reported that lactate and alanine were frequently altered in PC tissues [40]. Our finding of glycine upregulation is supported by Giskeodegard GF et al. [41], who studied the metabolome in prostate cancer tissue from a Spanish cohort by high resolution magic angle spinning magnetic resonance spectroscopy (HR-MAS). While glycine, L-lactate, and L-alanine have already been shown in literature, to the best knowledge of the authors, the present study for the first time describes guanidinoacetate and phenylacetylglycine as significant metabolites in PC [42].

More evidence for PC-specific metabolic alterations come from metabolomics studies in serum. Kumar et al. found by ^1^H-NMR that alanine, pyruvate, glycine, and sarcosine were significantly altered in serum of an Indian cohort of PC patients [43]. These results were supported by Miyagi et al., using high performance liquid chromatography-electrospray ionization mass spectrometry (HPLC-ESE-MS), showing a significant change of alanine, glutamine, valine, tryptophan, arginine and isoleucine, ornithine, and lysine levels associated with PC in a Japanese cohort [44]. However, while Kumar et al. found an upregulation of alanine [43], the alanine levels were downregulated in the study by Miyagi et al. [43]. The discrepancy of two studies showed that the different methods potentially may cause different findings [45].

Extensive literature survey revealed only few studies of urine metabolite levels in PC (Table 3). Only one study reported changes for two of the metabolites identified in our study: glycine and dimethylglycine. However, opposite to our results, those metabolites were downregulated in the study of Pérez-Rambla and colleagues [46]. No urine level data are available for the other metabolites that turned up significantly altered in our PC cohort.

The interpretation of these differences is difficult. Different compositions of the PC cohorts in respect to tumor stage may be one reason, as the majority of our samples were from patients with metastasis and high-grade tumors. In addition, the control cohort in the study of Pérez-Rambla et al., were BPH patients, which could possibly explain the different findings [46]. Only 36% (18/50) of our control patients were diagnosed with BPH and the expression levels of glycine and dimethylglycine were not significantly different between BPH and non-BPH patients (Figure S2). Furthermore, the studies listed in Table 3, were done in different populations. Caucasian population samples were from western countries, which not only have a different genetic background but also represent different lifestyle and diet [51]. The study populations of two other studies were from Chinese patients, as in our study, but used different methods. Moreover, the lifestyle and diet of the patients from northern and southern China may not be comparable to the urban population we studied. Therefore, the results might reflect a research method, ethnic peculiarity and/or lifestyle or diet impact [45,51].

### 4.2. Potential Biomarkers of PC

Over the past 30 years, NMR and MRSI (magnetic resonance spectroscopic imaging) as a non-invasive test, are continuous performed to identify predictive/prognostic metabolic marker of PC [52]. Furthermore, considerable efforts are ongoing to develop high precision, reliable, safe and non-invasive diagnosis strategies. Kumar proposed a great question: “Metabolomics-Derived Prostate Cancer Biomarkers: Fact or Fiction?” In fact, their findings confirmed that NMR-based serum metabolomics analysis is a promising method for probing PC [43].

Using serum metabolomics, Kumar et al. found that L-alanine, pyruvate, glycine, and sarcosine were able to accurately differentiate 90.2% of cancer cases from healthy persons, with high sensitivity (84.4%) and specificity (92.9%) [43]. Glycine alone showed an AUC of 0.817 [43]. In our ^1^H-NMR study, we found, that glycine in urine was up-regulated in PC, and ROC analysis revealed for glycine an AUC of 0.70 (sensitivity = 72%, specificity = 64%), which is comparable to the performance in serum. Furthermore, ROC analysis was also performed to evaluate the various combination; however, the best combination of guanidinoacetate, phenylacetylglycine, and glycine did not significantly improve the discriminant ability (AUC of 0.77, sensitivity = 80%, and specificity = 64%), but significantly improved the sensitivity. In essence, the ROC findings revealed that guanidinoacetate, phenylacetylglycine, and glycine were potential biomarkers.

### 4.3. Metabolite Interactions and Pathways Potentially Involved in PC

A better understanding of relative correlation and interaction of the potential biomarkers in urine could provide insights into the pathological progression of the disorder. Interestingly, we observed a strong positive correlation between guanidinoacetate and phenylacetylglycine (*r* = 0.93, *p*-value < 0.001), while only moderate positive correlation between guanidinoacetate and glycine, phenylacetylglycine, and glycine. Furthermore, the comprehensive network showed that the direct and indirect interactions between the prominent metabolites (Figure 7A). Thus, the above results probably indicated that these metabolites are conditioning each other through direct or intermediates interaction. 

Glycine is a nonessential amino acid with a central role in protein metabolism and also functions as inhibitory neurotransmitter in the central nervous system [53,54]. Additionally, glycine is involved in the body’s production of DNA and in the energy balance [55,56,57]. Notably, its role in the biosynthesis of purines and in mitochondrial oxidative phosphorylation has been recognized as driver of cancer initiation and proliferation [58,59]. The elevated glycine urine levels in PC support this view and could explain the higher guanidinoacetate levels measured. Guanidinoacetate is a direct metabolite of glycine formed by the glycine aminotransferase. Interestingly, guanidinoacetate is further methylated by the guanidinoacetate N-methyltransferase to creatine, which can be converted to creatinine, which also was elevated in our PC patients as a trend [60]. In addition, Kim et al. found an association of aberrant genes of the “Glycine, serine, and threonine metabolism” pathway with metastasis in PC [61]. Our results also support the notion of altered “Glycine, serine, and threonine metabolism” pathway in PC, and that two of the related metabolites, namely glycine and guanidinoacetate, are potential biomarkers for differentiation of PC from healthy controls.

Previous research described that phenylacetylglycine is working as an acyl glycine [45]. As we known, acyl glycines as classical minor metabolites are one kinds of fatty acids [45,62,63]. Together with phenylacetylglutamine and phenylalanine, phenylacetylglycine is a representative of the phenylalanine/tyrosine metabolism (KEGG: map00360) and showed significant association with T stage in gastric cancer [42,64]. Our study for the first time shows elevated levels of phenylacetylglycine in the urine of prostate cancer patients and thereby further supports the importance of the phenylalanine/tyrosine metabolic pathway in cancer.

### 4.4. The Major Findings of the Present Study

In summary, the present study identified five prominent metabolites: guanidinoacetate, phenylacetylglycine, glycine, l-lactate, and l-alanine. NMR-derived urinary metabolomics seem sufficiently robust to detect PC. In comparison with previous studies, the most interesting findings were

- The metabolites guanidinoacetate, phenylacetylglycine, and glycine were significantly upregulated in urine samples of PC. On the contrary, l-alanine and l-lactate were significantly downregulated. Furthermore, the majority of them were positively correlated. Especially strong correlations were seen between guanidinoacetate, phenylacetylglycine and glycine.
- Guanidinoacetate, phenylacetylglycine, and glycine urine levels were significantly different between PC patients stratified for low GS (≤6) and high GS (≥7).
- Using the network module, we comprehensively described the potential interaction between the most prominent metabolites. ROC analyses of prominent metabolites revealed a reasonably high diagnostic accuracy of guanidinoacetate, phenylacetylglycine, and glycine.
- Pathway enrichment analysis indicated “Glycine, Serine, and Threonine metabolism” as the most importantly altered pathway. Those results provide evidence for the metabolites, and associated pathway potentially playing an essential role in PC.
- Here, we reported for the first time that guanidinoacetate, and phenylacetylglycine could be promising novel urine biomarkers for PC.

The limitations of our study are (1) the cohort size is small, and we lack an external validation cohort; therefore, our results are at risk of overfitting; (2) as the aim of this study was to evaluate the performance of urine ^1^H-NMR metabolomics in an Asian cohort, we did not include Caucasian patients for comparison; (3) due to the small cohort we were not able to analyze PC subgroups, e.g., PSA/Gleason Score/Metastases; and (4) In addition, we focused on the metabolites in urine. Therefore, we cannot estimate the differences of discrimination ability between the blood sample, urine sample and tissue sample at the same time.

Further research will have to validate the urine metabolite biomarker panel in a larger cohort. Comparison to a matched Caucasian cohort could provide interesting insights into ethnical differences, which would have a severe impact on the clinical implementation of urine metabolomics biomarker in different populations.

## 5. Conclusions

Based on the metabolic profiling of urine, the present study showed that PC could be distinguished from non-cancerous individuals by guanidinoacetate, phenylacetylglycine, and glycine. The findings may add to our understanding of the basic mechanisms and progression of PC and indicated that these metabolites are potential candidate markers for PC. Moreover, the present study supported the view that urine metabolomics-derived biomarkers for PC can be a new option for non-invasive PC diagnostics.

## Figures and Tables

**Table 1:** Characteristics of the individuals.

Characteristics	Control Group (*n* = 50)		PC Group (*n* = 50)		Significance
	Mean (SD)	Group Size	Mean (SD)	Group Size	*p*-Value
Age (years)	63.30 (9.61)	50	70.00 (8.98)	50	<0.0001
Prostate volume (mL)	26.24 (8.77)	24	39.77(19.00)	50	0.0169
PSA (≤ 10 ng/mL)	1.56 (0.89)	50	6.69 (1.96)	14	
PSA (10.1–20 ng/mL)	NA	0	14.01 (2.08)	14	
PSA (> 20 ng/mL)	NA	0	89.82 (86.28)	22	
GS (pre) 6	NA	NA	NA	13	
GS (pre) ≥7	NA	NA	NA	34	
GS (post) 6	NA	NA	NA	6	
GS (post) ≥7	NA	NA	NA	35	
Treatment:				50	
Radical operation				41	
Seed implantation				5	
Endocrine				2	
Chemotherapy				1	
TURP				1	

GS = Gleason Score; GS (pre) = GS of biopsy; 41 patients have accepted radical operation and got the post-operation GS (GS (post)); SD = standard deviation; prostate volume was calculated as volume: volume (mL) = (length × width × height) × π/6. TURP = Transurethral resection of the prostate; NA = not applicable; PC = prostate cancer.

**Table 2:** Twenty identified metabolites.

Key	Metabolites	HMDB ID	Moieties	Chemical Shifts ^a^	VIP
1	L-lactate	HMDB0000190	αCH, βCH_3_	1.33 (d,J = 6.6Hz),4.13 (q,J = 4.8Hz)	1.43
2	L-alanine	HMDB0000161	βCH_3_	1.48 (d, J = 7.2Hz)	1.76
3	acetate	HMDB0000042	CH_3_	1.92 (s)	1.45
5	succinate	HMDB0000254	CH_2_	2.41 (s)	0.06
6	citrate	HMDB0000094	half CH_2_, half CH_2_	2.54 (d,J = 16.2 Hz), 2.70 (d, J = 15.6 Hz)	0.42
7	dimethylglycine	HMDB0000092	N-CH_3_, CH_2_	2.92 (s), 3.72 (s)	1.06
8	formate	HMDB0000142	CH	8.46 (s)	0.99
11	dimethylamine	HMDB0000087	CH_3_	2.73 (s)	0.82
12	methylguanidine	HMDB0001522	CH_3_	2.85 (s)	0.17
13	trimethylamine	HMDB0000906	CH_3_	2.88 (s)	0.89
14	creatinine	HMDB0000562	CH_3_, CH_2_	3.04 (s), 4.06 (s)	0.45
15	taurine	HMDB0000251	S-CH_2_, N-CH_2_	3.27 (t), 3.42 (t)	0.29
16	betaine	HMDB0000043	N(CH_3_)_3_, CH_2_	3.27 (s), 3.90 (s)	0.09
17	guanidinoacetate	HMDB0000128	CH_2_	3.80 (s)	1.94
18	hippurate	HMDB0000714	CH_2_, CH, CH, CH	3.97 (d,J = 6Hz), 7.55 (t,J = 7.8Hz), 7.64 (t,J = 7.8Hz), 7.84 (d,J = 7.2Hz)	0.02
19	N-methylnicotinamide	HMDB0003152	2-CH, 4-CH, 6-CH, 5-CH, CH_3_	9.29 (s), 8.97 (d,J = 6Hz), 8.91 (dt), 8.19 (m), 4.48 (s)	0.55
20	2-Hydroxyisobutyrate	HMDB0000729	CH_3_	1.36 (s)	0.36
21	glycine	HMDB0000123	CH_2_	3.57 (s)	1.36
22	fumaric acid	HMDB0000134	CH	6.56 (s)	0.32
28	Phenylacetylglycine	HMDB0000821	CH_2,_ CH, CH	3.68 (s), 7.37 (m), 7.43 (m)	1.59

^a^ Signal position in parts per million (ppm) in relation to TPS (set to 0 ppm).

**Table 3:** Metabolites studied in previous studies of Prostate cancer (PC).

Metabolites		Samples (Methods)	Reference	Ethnos
Up-Regulated	Down-Regulated			
BCAA, glutamate; pseudouridine	Glycine ^@^, dimethylglycine ^@^, fumarate, 4-imidazole-acetate	Urine (1H-NMR)	Pérez-Rambla et al. [46]	Spanish
glycocholic acid, hippurate, chenodeoxycholic acid	5-Hydroxy-l-tryptophan, taurocholic acid	Urine (FPLC/MS)	Liang, et al. [47]	Chinese (Northern of China)
	citrate, Myo-inositol, spermine	EPS (1H-NMR)	Serkova et al. [48]	American
sarcosine		Urine/PT/Plasma (GC-MS)	Sreekumr et al. [49]	American
propenoic acid, dihyroxybutanoic acid xylonic acid	pyrimidine, creatinine, purine, glucopyranoside, xylopyranoseand, ribofuranoside	Urine (GC-MS)	Wu et al. [50]	Chinese (Southern of China)

^@^ opposite to the present study; EPS: Human expressed prostatic secretions; BCCA: Branched-chain amino acids; PT: Prostate Tissue; GC-MS: Gas chromatography/mass spectrometry; FPLC/MS: Faster ultrahigh performance liquid chromatography-mass spectrometry; ^1^H-NMRS: Proton nuclear magnetic resonance spectroscopy.

## Data Availability

Data is contained within the article or supplementary material.

## Supplementary Materials

The following are available online at https://www.mdpi.com/2075-4418/11/2/149/s1, Figure S1: Data normalization, density and intensity before and after data normalization; Figure S2: glycine and dimethylglycine levels. Comparion between normal cases, BPH cases and cancer patients; difference are significant between normal and cancers, but not between BPH and normal cases; ANOVA, Tukey’s multiple comparison test, ** *p* < 0.01; **** *p* < 0.0001; Figure S3: subgroup analysis based on biopsy GS (GS (pre)); Figure S4: subgroup analysis based on radical prostatectomy GS (GS (post)); Figure S5: subgroup analysis based on PSA of PCa; stratification following the guidelines of the EAU [65]: PSA ≤ 10 ng/mL (*n* = 14), PSA 10.1–20 ng/mL (*n* = 14), PSA > 20 ng/mL (*n* =22); Figure S3 and Figure S5: data presented as box plots with scatter plot, line in box indicates mean, whiskers indicate 95% CI; Table S1: data cleansing of the combined data sets; Table S2: data cleansing of the cancer data set; Table S3: data cleansing of the control data set; Table S4: listing of the thirty metabolites analyzed; Table S5: tissue location and expression of the five most prominent metabolites; Table S6: metabolite detection by different models and variable reduction process; Table S7: Urine levels of the top 8 metabolites.

## Abbreviations

AUC	Area under the receiver operating characteristic (ROC) curve
CI	Confidence Interval
DRE	Digital rectal examination
FC	Fold change
GAA	Guanidinoacetate
GC/MS	Gas chromatography-mass spectrometry
GS	Gleason score
HMDB	Human Metabolome Database
NMR	Nuclear magnetic resonance
OPLS-DA	Orthogonal Partial Least Squares discriminant analysis
PBS	Phosphate buffer solution
PC	Prostate cancer
PCA	Principal component analysis
PLS-DA	Partial Least Squares discriminant analysis
PSA	Prostate specific antigen
ROC	Receiver operating characteristic curve
STITCH	Search tool for interactions of chemicals
TSP	Trimethylsilylpropionic acid-d_4_ sodium salt
TURP	Transurethral resection of the prostate
VIP	Variable importance in projection
