# Supplementary material for: Novel Metabolic Signatures of Prostate Cancer Revealed by 1H-NMR Metabolomics of Urine
Source: Diagnostics (Basel). 2021 Jan 20;11(2):149. doi: 10.3390/diagnostics11020149 (PMC7909529; doi:10.3390/diagnostics11020149)
Supplement: Supplementary file 1 [file diagnostics-11-00149-s001.zip › Figure S2.docx]

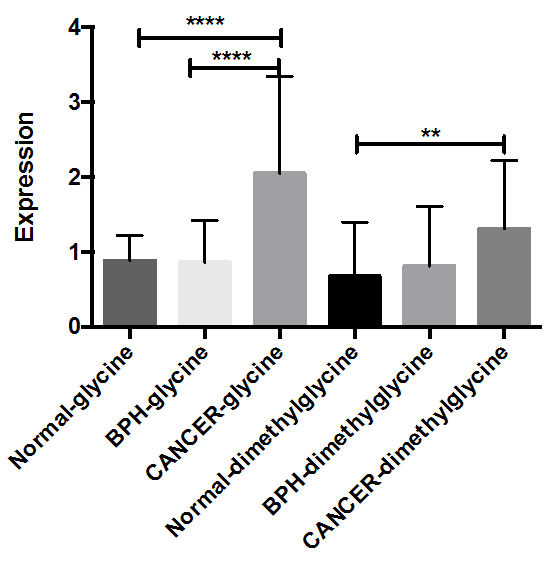


Figure S2: Glycine and dimethylglycine levels. Comparion between normal cases, BPH cases and cancer patients; differences are significant between normal and cancers, but not between BPH and normal cases; ANOVA, Tukey’s multiple comparison test, **p<0.01; ****p<0.0001.
