# Supplementary material for: Novel Metabolic Signatures of Prostate Cancer Revealed by 1H-NMR Metabolomics of Urine
Source: Diagnostics (Basel). 2021 Jan 20;11(2):149. doi: 10.3390/diagnostics11020149 (PMC7909529; doi:10.3390/diagnostics11020149)
Supplement: Supplementary file 1 [file diagnostics-11-00149-s001.zip › Figure S3.docx]

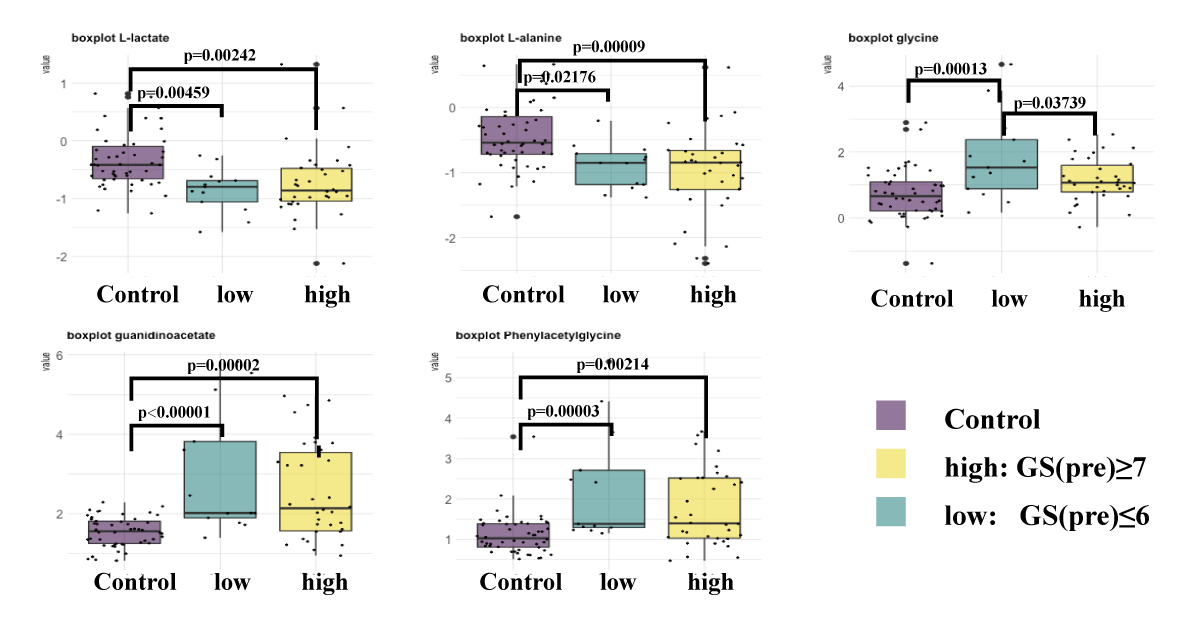


Figure S3: Subgroup analysis based on biopsy GS (GS(pre)). Data presented as box plots with scatter plot, line in box indicates mean, whiskers indicate 95% CI.
