# Supplementary material for: Novel Metabolic Signatures of Prostate Cancer Revealed by 1H-NMR Metabolomics of Urine
Source: Diagnostics (Basel). 2021 Jan 20;11(2):149. doi: 10.3390/diagnostics11020149 (PMC7909529; doi:10.3390/diagnostics11020149)
Supplement: Supplementary file 1 [file diagnostics-11-00149-s001.zip › Figure S5.docx]

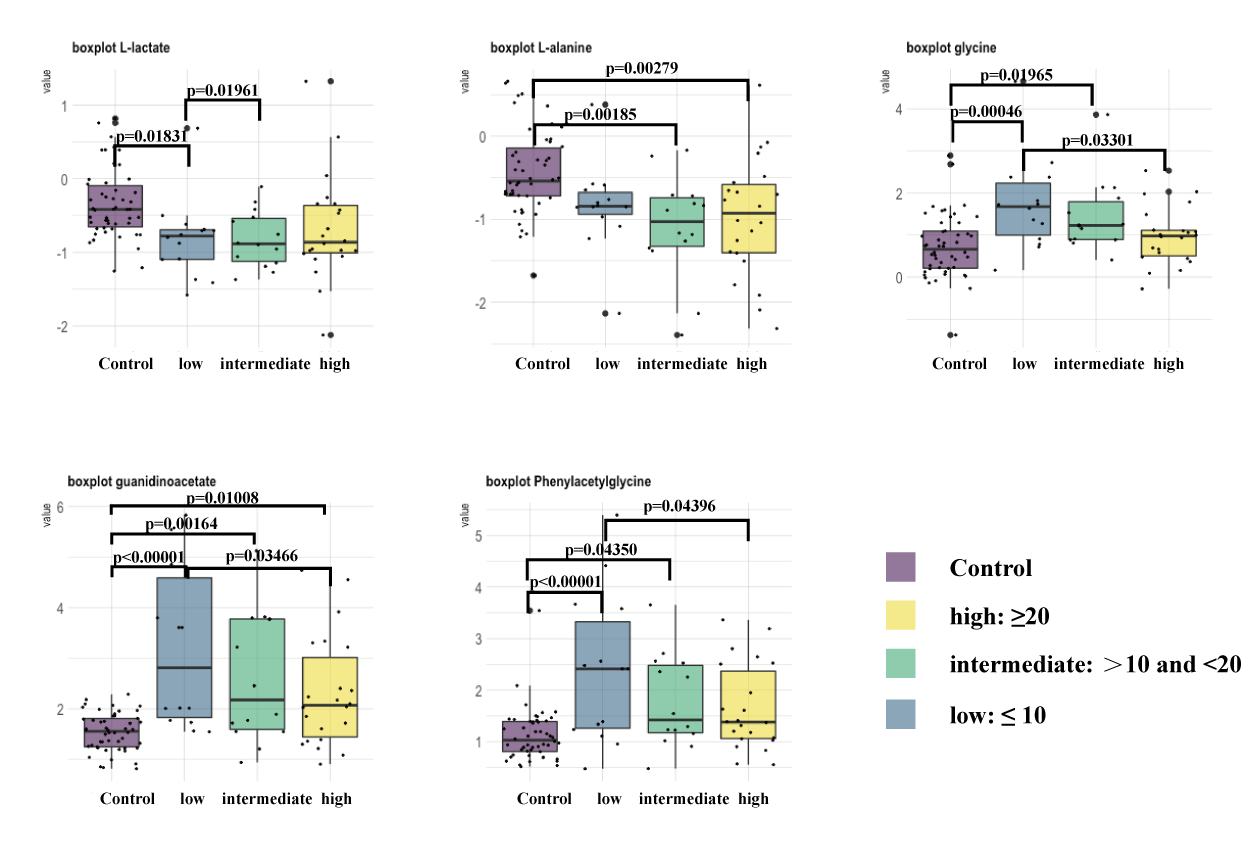


Figure S5: subgroup analysis based on PSA of PCa. Stratification following the guidelines of the EAU [65]: PSA ≤ 10 ng/ml (*n* = 14), PSA 10.1 – 20 ng/ml (*n* = 14), PSA > 20 ng/ml (*n* = 22); Figures S3-5: data presented as box plots with scatter plot, line in box indicates mean, whiskers indicate 95% CI.
