# Supplementary material for: Novel Metabolic Signatures of Prostate Cancer Revealed by 1H-NMR Metabolomics of Urine
Source: Diagnostics (Basel). 2021 Jan 20;11(2):149. doi: 10.3390/diagnostics11020149 (PMC7909529; doi:10.3390/diagnostics11020149)
Supplement: Supplementary file 1 [file diagnostics-11-00149-s001.zip › Table S1.docx]

Table S1: data cleansing of the combined data sets. Comparison of the data set variables before and after replacement of missing values, zeros and negative values by the 1/5 of the minimum positive value of each variable.

| Characteristic_compare_two data set | | | |
| --- | --- | --- | --- |
| **Metabolites Name** | **Before Replacement(data)** | **After Replacement(data_1)** | ***p*-value** |
| **Sample size** | 100 | 100 |  |
| **l-lactate (mean(sd))** | 0.97 (0.93) | 0.97 (0.93) | 1 |
| **l-alanine (mean(sd))** | 0.91 (0.93) | 0.91 (0.93) | 1 |
| **acetate (mean(sd))** | 0.98 (0.76) | 0.98 (0.76) | 1 |
| **succinate (mean(sd))** | 0.99 (2.25) | 0.99 (2.25) | 1 |
| **citrate (mean(sd))** | 7.33 (6.27) | 7.33 (6.27) | 1 |
| **dimethylglycine (mean(sd))** | 8.56 (21.39) | 8.56 (21.39) | 1 |
| **formate (mean(sd))** | 0.13 (0.08) | 0.13 (0.08) | 1 |
| **dimethylamine (mean(sd))** | 2.19 (1.77) | 2.19 (1.77) | 1 |
| **methylguanidine (mean(sd))** | 0.34 (0.27) | 0.34 (0.27) | 1 |
| **trimethylamine (mean(sd))** | 0.53 (0.42) | 0.53 (0.42) | 1 |
| **creatinine (mean(sd))** | 38.27 (28.98) | 38.27 (28.98) | 1 |
| **taurine (mean(sd))** | 5.82 (19.39) | 5.82 (19.39) | 1 |
| **betaine (mean(sd))** | 4.96 (15.07) | 4.96 (15.07) | 1 |
| **guanidinoacetate (mean(sd))** | 8.48 (17.28) | 8.48 (17.28) | 1 |
| **hippurate (mean(sd))** | 2.93 (3.93) | 2.93 (3.93) | 1 |
| **N-methylnicotinamide (mean(sd))** | 0.03 (0.04) | 0.03 (0.04) | 0.99 |
| **2-hydroxyisobutyrate (mean(sd))** | 0.73 (0.54) | 0.73 (0.54) | 1 |
| **glycine (mean(sd))** | 2.93 (4.47) | 2.93 (4.47) | 1 |
| **Fumaric.acid (mean(sd))** | 0.01 (0.02) | 0.01 (0.02) | 0.34 |
| **Phenylacetylglycine (mean(sd))** | 4.62 (7.57) | 4.62 (7.57) | 1 |
