# Supplementary material for: Novel Metabolic Signatures of Prostate Cancer Revealed by 1H-NMR Metabolomics of Urine
Source: Diagnostics (Basel). 2021 Jan 20;11(2):149. doi: 10.3390/diagnostics11020149 (PMC7909529; doi:10.3390/diagnostics11020149)
Supplement: Supplementary file 1 [file diagnostics-11-00149-s001.zip › Table S2.docx]

Table S2: data cleansing of the cancer data set. Comparison of the cancer data set variables before and after replacement of missing values, zeros and negative values by the 1/5 of the minimum positive value of each variable.

| Characteristic_compare_subgroup_cancer samples | | | |
| --- | --- | --- | --- |
| **Metabolites Name** | **Before Replacement(data)** | **After Replacement(data_1)** | ***p*-value** |
| **Sample size** | 50 | 50 |  |
| **l-lactate (mean(sd))** | 0.71 (0.7) | 0.71 (0.7) | 1 |
| **l-alanine (mean(sd))** | 0.64 (0.63) | 0.64 (0.63) | 1 |
| **acetate (mean(sd))** | 0.74 (0.57) | 0.74 (0.57) | 1 |
| **succinate (mean(sd))** | 1.05 (3.14) | 1.05 (3.14) | 1 |
| **citrate (mean(sd))** | 6.32 (4.95) | 6.32 (4.95) | 1 |
| **dimethylglycine (mean(sd))** | 7.68 (15.47) | 7.68 (15.47) | 1 |
| **formate (mean(sd))** | 0.12 (0.08) | 0.12 (0.08) | 1 |
| **dimethylamine (mean(sd))** | 1.76 (1.52) | 1.76 (1.52) | 1 |
| **methylguanidine (mean(sd))** | 0.29 (0.23) | 0.29 (0.23) | 1 |
| **trimethylamine (mean(sd))** | 0.42 (0.31) | 0.42 (0.31) | 1 |
| **creatinine (mean(sd))** | 30.79 (21.14) | 30.79 (21.14) | 1 |
| **taurine (mean(sd))** | 3.74 (12.97) | 3.74 (12.97) | 1 |
| **betaine (mean(sd))** | 3.98 (11.07) | 3.98 (11.07) | 1 |
| **guanidinoacetate (mean(sd))** | 12.91 (23.59) | 12.91 (23.59) | 1 |
| **hippurate (mean(sd))** | 3.15 (5.31) | 3.15 (5.31) | 1 |
| **N-methylnicotinamide (mean(sd))** | 0.02 (0.03) | 0.02 (0.03) | 0.98 |
| **2-hydroxyisobutyrate (mean(sd))** | 0.63 (0.49) | 0.63 (0.49) | 1 |
| **glycine (mean(sd))** | 3.69 (6.17) | 3.69 (6.17) | 1 |
| **Fumaric.acid (mean(sd))** | 0 (0) | 0 (0) | 0.56 |
| **Phenylacetylglycine (mean(sd))** | 6.33 (10.34) | 6.33 (10.34) | 1 |
