# Supplementary material for: Novel Metabolic Signatures of Prostate Cancer Revealed by 1H-NMR Metabolomics of Urine
Source: Diagnostics (Basel). 2021 Jan 20;11(2):149. doi: 10.3390/diagnostics11020149 (PMC7909529; doi:10.3390/diagnostics11020149)
Supplement: Supplementary file 1 [file diagnostics-11-00149-s001.zip › Table S3.docx]

Table S3: data cleansing of the control data set. Comparison of the control data set variables before and after replacement of missing values, zeros and negative values by the 1/5 of the minimum positive value of each variable.

| Characteristic_compare_subgroup_control samples | | | |
| --- | --- | --- | --- |
| **Metabolites Name** | **Before Replacement(data)** | **After Replacement(data_1)** | **P-value** |
| **Sample size** | 50 | 50 |  |
| **L-lactate (mean(sd))** | 1.23 (1.07) | 1.23 (1.07) | 1 |
| **L-alanine (mean(sd))** | 1.18 (1.1) | 1.18 (1.1) | 1 |
| **acetate (mean(sd))** | 1.22 (0.85) | 1.22 (0.85) | 1 |
| **succinate (mean(sd))** | 0.93 (0.58) | 0.93 (0.58) | 1 |
| **citrate (mean(sd))** | 8.34 (7.26) | 8.34 (7.26) | 1 |
| **dimethylglycine (mean(sd))** | 9.45 (26.14) | 9.45 (26.14) | 1 |
| **formate (mean(sd))** | 0.13 (0.09) | 0.13 (0.09) | 1 |
| **dimethylamine (mean(sd))** | 2.63 (1.9) | 2.63 (1.9) | 1 |
| **methylguanidine (mean(sd))** | 0.39 (0.3) | 0.39 (0.3) | 1 |
| **trimethylamine (mean(sd))** | 0.65 (0.48) | 0.65 (0.48) | 1 |
| **creatinine (mean(sd))** | 45.74 (33.69) | 45.74 (33.69) | 1 |
| **taurine (mean(sd))** | 7.89 (24.13) | 7.89 (24.13) | 1 |
| **betaine (mean(sd))** | 5.95 (18.28) | 5.95 (18.28) | 1 |
| **guanidinoacetate (mean(sd))** | 4.06 (2.6) | 4.06 (2.6) | 1 |
| **hippurate (mean(sd))** | 2.7 (1.71) | 2.7 (1.71) | 1 |
| **N-methylnicotinamide (mean(sd))** | 0.03 (0.04) | 0.03 (0.04) | 1 |
| **2-hydroxyisobutyrate (mean(sd))** | 0.83 (0.57) | 0.83 (0.57) | 1 |
| **glycine (mean(sd))** | 2.16 (1.1) | 2.16 (1.1) | 1 |
| **Fumaric.acid (mean(sd))** | 0.01 (0.03) | 0.02 (0.03) | 0.44 |
| **Phenylacetylglycine (mean(sd))** | 2.91 (1.69) | 2.91 (1.69) | 1 |
