# Supplementary material for: Novel Metabolic Signatures of Prostate Cancer Revealed by 1H-NMR Metabolomics of Urine
Source: Diagnostics (Basel). 2021 Jan 20;11(2):149. doi: 10.3390/diagnostics11020149 (PMC7909529; doi:10.3390/diagnostics11020149)
Supplement: Supplementary file 1 [file diagnostics-11-00149-s001.zip › Table S4.docx]

Table S4: listing of the thirty metabolites analyzed

| Key | Metabolites | HMDB ID | Moieties | Chemical shifts ^a^ |
| --- | --- | --- | --- | --- |
| 1 | L-Lactate | HMDB0000190 | αCH, βCH_3_ | 1.33(d,J=6.6Hz), 4.13(q,J=4.8Hz) |
| 2 | L-Alanine | HMDB0000161 | βCH_3_ | 1.48(d, J=7.2Hz) |
| 3 | acetate | HMDB0000042 | CH_3_ | 1.92(s) |
| 4 | pyruvate | HMDB0000243 | CH_3_ | 2.37(s) |
| 5 | succinate | HMDB0000254 | CH_2_ | 2.41(s) |
| 6 | citrate | HMDB0000094 | half CH_2_, half CH_2_ | 2.54(d,J=16.2 Hz), 2.70(d, J=15.6 Hz) |
| 7 | dimethylglycine | HMDB0000092 | N-CH_3_, CH_2_ | 2.92(s), 3.72(s) |
| 8 | formate | HMDB0000142 | CH | 8.46(s) |
| 9 | pantothenate | HMDB0000210 | CH_3_, CH_3_, CHOH | 0.94(s), 0.90(s), 4.02(s) |
| 10 | methylamine | HMDB0000164 | CH_3_ | 2.61(s) |
| 11 | dimethylamine | HMDB0000087 | CH_3_ | 2.73(s) |
| 12 | methylguanidine | HMDB0001522 | CH_3_ | 2.85(s) |
| 13 | trimethylamine | HMDB0000906 | CH_3_ | 2.88(s) |
| 14 | creatinine | HMDB0000562 | CH_3_, CH_2_ | 3.04(s), 4.06(s) |
| 15 | taurine | HMDB0000251 | S-CH_2_, N-CH_2_ | 3.27(t), 3.42(t) |
| 16 | betaine | HMDB0000043 | N(CH_3_)_3_, CH_2_ | 3.27(s), 3.90(s) |
| 17 | guanidinoacetate | HMDB0000128 | CH_2_ | 3.80(s) |
| 18 | hippurate | HMDB0000714 | CH_2_, CH, CH, CH | 3.97(d,J=6Hz), 7.55(t,J=7.8Hz), 7.64(t,J=7.8Hz), 7.84(d,J=7.2Hz) |
| 19 | N-methylnicotinamide | HMDB0003152 | 2-CH, 4-CH, 6-CH, 5-CH, CH_3_ | 9.29(s), 8.97(d,J= 6Hz), 8.91(dt),  8.19(m), 4.48(s) |
| 20 | 2-Hydroxyisobutyrate | HMDB0000729 |  | 1.36(s) |
| 21 | glycine | HMDB0000123 |  | 3.57(s) |
| 22 | fumaric acid | HMDB0000134 |  | 6.56(s) |
| 23 | lipid | HMDB0013244 |  | 0.89(m), 1.27(m), 2.0(m) |
| 24 | leucine | HMDB0000687 |  | 0.96(d, 6.6) |
| 25 | acetamide | HMDB0031645 |  | 1.99(s) |
| 26 | malonic acid | HMDB0000691 |  | 3.15(s) |
| 27 | tMAO | HMDB0000925 |  | 3.27(s) |
| 28 | phenylacetylglycine | HMDB0000821 |  | 3.68(s), 7.37(m), 7.43(m) |
| 29 | uridine | HMDB0000296 |  | 4.30(t, 5.4), 7.88(d,5.4) |
| 30 | adenosine | HMDB0000050 |  | 8.34 (s), 8.22 (s) |

*VIP means Variable importance in the projection; Key number was related to the number in Figure 1.
