# Supplementary material for: Novel Metabolic Signatures of Prostate Cancer Revealed by 1H-NMR Metabolomics of Urine
Source: Diagnostics (Basel). 2021 Jan 20;11(2):149. doi: 10.3390/diagnostics11020149 (PMC7909529; doi:10.3390/diagnostics11020149)
Supplement: Supplementary file 1 [file diagnostics-11-00149-s001.zip › Table S5.docx]

Table S5: tissue location and expression of the five most prominent metabolites

| Metabolites | Chemical Formula | Prostate location | HMDB code | KEGG ID |
| --- | --- | --- | --- | --- |
| guanidinoacetate | C_3_H_7_N_3_O_2_ | no | HMDB0000128 | C00581 |
| phenylacetylglycine | C_10_H_11_NO_3_ | unknow | HMDB0000821 | C05598 |
| glycine | C_2_H_5_NO_2_ | yes | HMDB0000123 | C00037 |
| L-lactate | C_3_H_6_O_3_ | yes | HMDB0000190 | C00256 |
| L-alanine | C_3_H_7_NO_2_ | yes | HMDB0000161 | C00041 |
