# Supplementary material for: Novel Metabolic Signatures of Prostate Cancer Revealed by 1H-NMR Metabolomics of Urine
Source: Diagnostics (Basel). 2021 Jan 20;11(2):149. doi: 10.3390/diagnostics11020149 (PMC7909529; doi:10.3390/diagnostics11020149)
Supplement: Supplementary file 1 [file diagnostics-11-00149-s001.zip › Table S7.pdf]

Table S7: Urine levels of the top 8 metabolites

Sample	Class	Age	BMI	PSA before Biopsy	Gleason pattern of biopsy	GS(pre)	Glesaon pattern of RPE	GS(post)	TNM staging	Risk stratification (EAU 2014) [1]	L-lactate	L-alanine	acetate	dimethylglycine	formate	guanidinoacetate	glycine	phenylacetylglycine
P1	patient	72	19.53	4.96	4+3	7	3+4	7	T2N0M0	2	-1.087593673	-1.086602737	0.2214996	0.396829152	-2.682630315	1.732039095	1.271356216	1.105773463
P2	patient	70	26.83	12.23	3+3	6	3+4	7	T2cN0M0	2	-0.893634074	-1.167064497	-0.789811384	0.78357574	-2.227598366	1.718865708	0.884517874	1.153931999
P3	patient	81	23.03	16.28	4+4	8	na	na	T2cN0M0	2	-0.414269369	-0.170468011	-0.190978928	0.277597779	-2.823905551	1.770713345	0.811011164	1.54536804
P4	patient	64	23.05	154	5+4	9	5+4	9	T2cN0M1	3	-0.777104299	-0.842758317	-0.5986936	3.708103595	-3.895360496	2.363296379	1.060273966	1.386809422
P5	patient	63	20.28	8.73	3+3	6	3+4	7	T3aN0M0	3	-0.702100148	-0.851156633	-0.847142536	2.598682881	-2.764582626	3.609315175	2.37445548	2.412339319
P6	patient	62	28.52	9.01	4+3	7	3+4	7	T2aN0M0	2	-0.702100148	-0.851156633	-0.847142536	2.598682881	-2.764582626	3.609315175	2.37445548	2.412339319
P7	patient	71	23.44	139.61	4+3	7	4+3	7	T3aN0M0	3	-1.525735735	-2.092788686	-1.149943257	3.106784058	-3.352564644	4.73760804	2.029819437	3.363600391
P8	patient	73	24.8	16.66	4+3	7	5+4	9	T4N1M0	3	-1.270129718	-0.834630326	-0.508622339	1.442014731	-3.226657626	1.208571158	1.157273456	0.90649507
P9	patient	61	26.62	14.11	4+4	8	3+4	7	T3aN0M0	3	-0.577816711	-0.812079208	-0.60728043	2.273562602	-2.710165311	3.763870977	1.257719928	2.35609889
P10	patient	88	18.73	>100	3+4	7	na	na	T2cN0M0	3	-0.339021069	-0.130210806	-0.132987541	0.945561627	-2.943552539	1.362060331	0.158660879	1.027096665
P11	patient	75	20.07	3.87	3+4	7	3+3	6	T2N0M0	2	-0.497864981	-0.834533911	-0.986917016	3.531328079	-5.097660289	4.855752869	1.816248543	3.6677696
P12	patient	63	25.56	15.15	4+3	7	3+5	8	T4N0M0	3	-1.143048522	-2.39520758	-1.343324971	3.397171673	-3.454270197	3.781586825	2.125631468	2.523934347
P13	patient	89	22.46	61.94	3+5	8	na	na	T2N0M0	3	-0.971449596	-0.769565135	-0.406398072	0.70220153	-5.011988807	1.605776923	0.664019104	1.410558365
P14	patient	51	27.06	11.16	3+3	6	3+4	7	T3aN0M0	3	-1.056577447	-1.381589129	-1.228033499	3.331638092	-3.747801569	3.820569503	3.861218292	2.711706184
P15	patient	61	31.84	39.133	5+4	9	5+4	9	T3bN1M0	3	-0.952715873	-1.255809814	-0.998916217	2.412620771	-2.911263111	2.173100832	1.48499338	1.177650221
P16	patient	70	23.46	>100	4+5	9	na	na	T4N1M1	3	-0.421802089	-0.075321978	-0.186173416	0.190602786	-3.310167348	1.086793995	0.090122869	0.832744022
P17	patient	70	21.47	8.51	3+3	6	3+5	8	T2aN0M0	1	-1.410849318	-1.234516016	-1.213848642	3.87929843	-6.288249891	5.83174701	2.720121952	4.416888114
P18	patient	72	28.34	14.37	3+3	6	3+5	8	T2bN0M0	2	-1.187742105	-1.347402093	-0.969758729	3.308570958	-3.27208139	5.126335533	1.529545515	3.655258402
P19	patient	77	20.28	5.585	4+3	7	4+4	8	T2aN0M0	2	-0.874324039	-0.575759632	-0.556575952	2.397477748	-2.925697408	1.54640041	0.781111002	0.953532968
P20	patient	81	25.61	14.09	3+5	8	na	na	T2aN0M0	3	-0.954831415	-0.89223565	-0.080672423	0.379848874	-3.557568571	1.551885717	1.214144847	1.230752401
P21	patient	78	19.03	6.893	3+3	6	3+3	6	T3aN0M0	3	-0.758152732	-0.855316801	0.472069613	1.100508012	-4.523205521	5.546097796	4.655619413	5.398041313
P22	patient	81	23.14	22.63	4+5	9	3+5	8	T3bN1M0	3	-1.01479225	-1.503924942	-0.40066083	2.411951131	-3.004274437	1.851929448	0.959426881	1.201758586
P23	patient	71	23.88	392.5	na	na	4+5	9	T4N1M1	3	-1.26633011	-1.791461181	-0.296603453	0.393596495	-2.322372714	0.908674346	0.442472397	1.630090887
P24	patient	84	27.61	140.52	5+4	9	na	na	T4N1M1	3	-0.842986938	-1.392192729	-0.47601657	1.11115598	-1.719862074	2.02743459	0.583969441	2.525337739
P25	patient	71	18.37	86.03	4+5	9	4+5	9	T3aN0M0	3	-0.97547815	-1.015287975	-0.619198734	3.2596953	-2.914970161	3.305081008	0.693052131	2.646439892
P26	patient	45	31.18	>154	4+5	9	4+3	7	T4N0M0	3	-0.467243782	-0.700734145	-0.376033626	0.969971093	-2.131625341	1.215467349	-0.279422662	0.552973956
P27	patient	67	31.18	181.6	4+5	9	4+5	9	T4N0M0	3	-0.976396128	-1.042878494	-1.026195634	3.098715107	-3.639799139	4.555410572	1.980801682	3.192858873
P28	patient	72	25.35	64.89	3+4	7	3+5	8	T4N0M0	3	0.568789387	0.613759247	-0.507806263	6.324632768	-3.420835812	2.405329624	0.366697322	1.056454786
P29	patient	84	22.49	23.76	3+3	6	na	na	T2aN0M0	3	-0.253820656	-0.206021306	-0.12071366	-0.066314746	-2.962791766	1.392555505	0.480482297	1.315392761
P30	patient	64	20.15	25.234	3+4	7	3+3	6	T3aN0M0	3	-0.677981282	-0.654210831	-0.854602996	2.995734449	-3.925703518	3.916872689	1.103654529	2.803787392
P31	patient	55	19.38	10.9	4+4	8	4+4	8	T2cN0M0	3	-0.753662175	-1.26356385	-0.937107637	2.480065347	-2.365069891	3.219564741	2.135139472	2.25332229
P32	patient	64	25.86	5.6	na	na	na	na	T1cN0M0	1	0.687792455	0.380056231	-0.482619061	2.163126341	-2.446468724	1.561208592	1.736728854	0.472968732
P33	patient	68	24.77	8.54	3+4	7	3+4	7	T3aN0M0	3	-1.096870511	-0.971223786	-0.603165276	3.168289132	-3.602051179	4.967245371	1.633892101	3.579612252
P34	patient	64	20.62	8.91	3+3	6	3+4	7	T2aN0M0	1	-0.616386441	-0.588739258	-0.295952786	3.476814935	-2.861573614	2.006903847	0.166303902	2.478711535
P35	patient	73	24.5	25.16	4+4	8	4+5	9	T4N0M0	3	1.326191183	-0.674562096	-0.793046393	2.664480806	-2.381884926	3.217781792	1.784494371	1.949764627
P36	patient	68	23.38	20.7	4+3	7	4+5	9	T3aN0M0	3	-0.338273494	-0.485304397	-0.055182056	3.098574154	-2.745470615	2.237241892	0.934205445	1.370288564
P37	patient	74	23.67	15.36	na	na	5+3	8	T3aN0M0	3	-1.367218048	-2.134729564	-1.320298662	2.148308569	-3.179333621	3.799354061	0.90541316	2.561993546
P38	patient	62	21.98	7.82	3+4	7	3+4	7	T3aN0M0	3	-1.367218048	-2.134729564	-1.320298662	2.148308569	-3.179333621	3.799354061	0.90541316	2.561993546
P39	patient	67	24.16	129.5	3+5	8	4+3	7	T3aN0M0	3	-1.05115207	-0.557784785	-0.517397123	2.008681512	-3.218763792	3.339654914	2.532665606	2.507058362
P40	patient	80	24.08	10.37	4+5	9	na	na	T2aN0M0	3	-0.521782087	-0.719288765	-0.238971414	0.729910938	-0.848004245	1.547812501	0.408043934	1.017380843
P41	patient	64	29.38	36.75	5+4	9	5+4	9	T4N0M0	3	-1.089550175	-1.141284687	-0.785199298	1.683847594	-2.635169332	1.298937318	1.080558023	0.569459099
P42	patient	59	23.78	13.88	3+3	6	3+5	8	T2aN0M0	2	-0.315135252	-0.709671332	-0.588624066	3.393335456	-2.019507404	1.893661808	1.87684754	1.223506385
P43	patient	77	20.38	20.441	4+4	8	3+5	8	T2aN0M0	3	-2.122392986	-2.315902786	-0.131117954	1.822459736	-2.92643954	2.041711309	0.997919172	1.608228367
P44	patient	69	26.93	16.28	3+4	7	5+3	8	T3bN0M0	3	-0.105868915	-0.237779196	0.361819462	0.284777583	-3.081361331	0.942696432	0.887066353	0.475979411
P45	patient	83	19.38	20.27	5+3	8	5+4	9	T4N0M0	3	0.042297428	-1.914849902	0.410268603	0.857098814	-2.956168954	2.101139791	1.012286589	1.076594001
P46	patient	74	23.89	15.27	3+3	6	4+3	7	T2aN0M0	2	-0.870462405	-1.184498471	-0.660514632	0.692189195	-2.567969119	2.458504983	1.239083361	1.295313899
P47	patient	64	22.78	7.06	3+3	6	3+3	6	T3aN0M0	3	-1.578443673	-0.649180596	-0.434854245	1.702601918	-2.86164207	2.017286332	1.36293973	1.385962761
P48	patient	65	26.73	3.58	3+3	6	3+3	6	T2aN0M0	3	-0.795493813	-0.802128489	0.318795748	1.832180285	-2.897024072	2.013085683	1.719862273	1.337312774
P49	patient	69	22.98	4.65	3+3	6	3+3	6	T1cN0M0	1	-0.687273777	-0.76333063	-0.412416131	2.267891765	-3.106050232	1.770164213	0.718877077	1.235113405
P50	patient	70	26.12	37.42	4+3	7	5+3	8	T2bN0M0	3	-0.8793356	-1.408482705	-0.834387768	0.760486071	-2.128565499	1.718183392	1.118142224	0.901884277
Co1	control	70	na	control	control	control	control	control	control	control	-0.758298865	-1.177861357	-0.779492732	0.92793533	-3.216109949	1.96933311	0.733829308	1.715068755
Co2	control	50	na	control	control	control	control	control	control	control	-0.287067321	0.148299026	0.131143167	2.591411568	-4.513163059	2.032472315	-0.017234652	1.24717899
Co3	control	60	na	control	control	control	control	control	control	control	0.391799207	0.402632879	-0.42146461	4.9753649	-4.842566316	1.602597705	-1.377421606	0.730311831
Co4	control	51	na	control	control	control	control	control	control	control	-1.25327545	-1.678075044	-0.052985616	1.051017172	-3.083918703	1.232877492	0.234301055	0.537311643
Co5	control	62	na	control	control	control	control	control	control	control	-0.613429444	-1.213420072	-0.317429853	1.163295458	-3.339854849	1.531718393	0.739681219	1.464270716
Co6	control	56	na	control	control	control	control	control	control	control	-0.759948438	-0.696425467	-0.614320281	1.434680342	-3.583993598	2.287652136	1.600122794	1.564283678
Co7	control	54	na	control	control	control	control	control	control	control	0.431080095	0.490199768	-0.798213847	2.428889125	-2.945283713	1.773971684	2.683910845	0.623903936
Co8	control	66	na	control	control	control	control	control	control	control	-0.404734294	-0.65888063	-0.752481401	2.824465997	-4.424828191	1.348710869	1.440298192	1.082805707
Co9	control	76	na	control	control	control	control	control	control	control	-0.411011932	-0.556799132	-0.362707552	0.470346364	-3.877990979	1.418341369	0.766488068	0.935866535
Co10	control	61	na	control	control	control	control	control	control	control	-0.268974983	-0.404703157	0.247593246	0.07467412	-3.816039214	0.993413227	-0.086349289	0.806390335
Co11	control	76	na	control	control	control	control	control	control	control	-0.520756599	-0.808998849	-0.228074913	0.344526439	-3.512119221	0.839171742	0.825883221	0.929725007
Co12	control	63	na	control	control	control	control	control	control	control	0.392563204	-0.125882248	-0.13871306	0.676796585	-3.334632233	2.095835741	0.971886343	1.381832903
Co13	control	61	na	control	control	control	control	control	control	control	0.760511249	0.660920001	-0.41644197	1.705904052	-3.414279864	1.225578789	0.210234157	0.622581588
Co14	control	77	na	control	control	control	control	control	control	control	-0.185985493	-0.264022472	0.691136456	0.956542869	-4.096124018	1.529893364	0.480357482	2.08767205
Co15	control	51	na	control	control	control	control	control	control	control	-0.523047682	-0.942222315	0.133235948	0.909059643	-3.105477962	1.989277287	1.707112398	1.193849703
Co16	control	63	na	control	control	control	control	control	control	control	-0.865480386	-0.563436634	-0.411748124	0.489679053	-3.194474705	1.862700844	1.50385368	0.701658955
Co17	control	58	na	control	control	control	control	control	control	control	-1.206537783	-1.138701583	-0.52258554	0.717932875	-3.279199132	1.605222502	0.273855349	1.108251802
Co18	control	56	na	control	control	control	control	control	control	control	-0.528707718	-0.886093194	-0.338929586	1.628448518	-3.534557686	1.350803658	0.74711062	1.107537393
Co19	control	54	na	control	control	control	control	control	control	control	-0.245286147	-0.562814138	-0.138368059	-0.077829394	-2.745730011	1.219819559	0.489034036	0.934610676
Co20	control	58	na	control	control	control	control	control	control	control	-0.388635135	-0.347109394	-0.445963598	-0.013120724	-4.795378938	1.244531806	-0.137386494	0.548028434
Co21	control	57	na	control	control	control	control	control	control	control	0.570217689	-0.926351463	-0.361438901	0.550598948	-3.614164824	2.18429381	0.416727812	0.887042962
Co22	control	51	na	control	control	control	control	control	control	control	-0.413337577	-0.57435326	0.408146249	0.397514161	-2.234950455	1.904678938	1.576038262	3.542661639
Co23	control	59	na	control	control	control	control	control	control	control	-0.591434864	-0.511289102	-0.073094204	0.316980669	-3.1115953	1.738584568	0.224413513	0.844823995
Co24	control	55	na	control	control	control	control	control	control	control	-0.416710128	-0.524280607	-0.313185095	0.057945432	-3.266450651	0.815305916	0.521557902	0.51443979
Co25	control	91	na	control	control	control	control	control	control	control	0.819433223	-0.285334261	-0.363738516	0.345164471	-3.999970709	1.166925537	0.043752892	1.577752476
Co28	control	64	na	control	control	control	control	control	control	control	0.394667569	0.50895341	-0.158785967	0.952248604	-2.720423872	1.618791831	2.889803314	0.817408891
Co29	control	50	na	control	control	control	control	control	control	control	-0.007844902	-0.065792483	-0.254482616	1.208466933	-3.594872497	1.496913481	0.97637755	0.971788825
Co30	control	56	na	control	control	control	control	control	control	control	-0.562417462	-0.194533936	0.182939801	0.160350211	-2.504767843	0.913072656	1.292678589	0.903979269
Co31	control	54	na	control	control	control	control	control	control	control	-0.532964012	-0.741880717	-0.358803894	6.154745367	-3.357314443	2.056184731	0.019174094	0.887007667
Co32	control	63	na	control	control	control	control	control	control	control	-0.751422589	-0.700047264	-0.401412652	1.760466846	-3.331045016	1.356367971	0.344051678	0.66179685
Co33	control	50	na	control	control	control	control	control	control	control	-0.601735405	-0.29333372	0.72377247	0.084259087	-2.909156645	1.256975341	1.00034134	1.192317967
Co34	control	55	na	control	control	control	control	control	control	control	0.191832953	0.639005436	0.123661273	3.352069686	-3.096397246	1.524866819	0.127027989	0.614022401
Co35	control	62	na	control	control	control	control	control	control	control	-0.07133405	0.08449125	-0.369118211	0.570949662	-2.227935265	1.579765913	1.520008071	0.74329995
Co36	control	67	na	control	control	control	control	control	control	control	-0.055511999	0.363590707	-0.069858773	3.033031285	-3.282847563	1.813445034	1.43400392	1.378187677
Co37	control	61	na	control	control	control	control	control	control	control	-0.28539859	-0.50551232	-0.139054472	0.892296758	-4.461790358	1.369770256	0.198370703	1.150845783
Co38	control	64	na	control	control	control	control	control	control	control	-0.606681522	-0.916166226	-0.134985708	0.95213488	-4.300989349	1.752418778	0.58910103	1.486992112
Co39	control	66	na	control	control	control	control	control	control	control	-0.680609278	-0.905983323	-0.170209745	0.53070287	-2.615578887	1.277249356	0.441059877	0.689452337
Co40	control	65	na	control	control	control	control	control	control	control	-0.950843144	-0.689044891	-0.059095049	4.246958672	-3.855938265	1.713259504	0.129659016	1.413977471
Co41	control	77	na	control	control	control	control	control	control	control	-0.6760086	-0.666885186	-0.5560817	1.168687163	-3.716901723	1.198425397	1.291113703	1.055091791
Co42	control	67	na	control	control	control	control	control	control	control	-0.002822484	0.10930192	-0.685654217	0.361411735	-3.301111265	1.318592499	0.066607829	0.697457305
Co43	control	63	na	control	control	control	control	control	control	control	-0.313810825	-0.587090462	-0.247296357	0.799579344	-3.05633813	2.069975066	0.881568823	1.390894477
Co44	control	65	na	control	control	control	control	control	control	control	-0.833018051	-0.714690837	-1.287518967	1.180674582	-3.323524927	1.797411313	1.027476356	1.42980226
Co45	control	60	na	control	control	control	control	control	control	control	-0.156543428	-0.04378647	-0.198432445	0.464230328	-3.214899757	1.585197415	0.811840528	1.004944411
Co46	control	74	na	control	control	control	control	control	control	control	-0.425905971	-0.414712261	-0.045131821	0.391301911	-3.833239622	1.181286168	0.585931997	0.946564889
Co47	control	83	na	control	control	control	control	control	control	control	-0.204172449	-0.232160776	-0.255306505	5.372406936	-3.486806933	1.860147321	0.536838284	1.266134035
Co48	control	71	na	control	control	control	control	control	control	control	-0.723579198	-1.061448843	-1.132343261	1.988001042	-2.864831097	1.73643569	1.083835044	1.360109722
Co49	control	81	na	control	control	control	control	control	control	control	-0.66746588	-0.693189635	-0.471050519	1.078338344	-4.143706652	1.958101371	0.048513884	1.502032353
Co50	control	83	na	control	control	control	control	control	control	control	0.209615445	-0.035958925	-0.288302034	0.464712222	-3.981526407	1.041630029	1.6822341	0.843069943
Co51	control	65	na	control	control	control	control	control	control	control	-0.568010557	-0.307005712	0.120356006	0.552978689	-2.850039626	0.854990547	-0.269139427	1.400489856
Co52	control	63	na	control	control	control	control	control	control	control	-0.791774338	-0.720345628	-0.428856543	0.519515957	-3.526338189	1.594447297	1.099543113	1.438410485

GS(pre) = Gleason Score of biopsy
GS(post) = final Gleason Score after radical prostatectomy (RPE)
BMI = body mass index

Stratification (EAU guidelines 2014)

1 low risk: PSA <=10 ng/ml **AND** preGS = 6 **AND** cT1c-cT2a
2 intermediate risk: PSA 10.1-20 ng/ml **OR** preGS =7 **OR** cT2b-c
3 high: >PSA 20 ng/ml **OR** preGS 8-10 **OR** >=cT3a

[1] Heidenreich et al. EAU guidelines on prostate cancer. part 1: screening, diagnosis, and local treatment with curative intent-update 2013. *Eur Urol* **2014**;*65*:124-37.
