# Supplementary figures and images for: Novel Metabolic Signatures of Prostate Cancer Revealed by 1H-NMR Metabolomics of Urine
Source: Diagnostics (Basel). 2021 Jan 20;11(2):149. doi: 10.3390/diagnostics11020149 (PMC7909529; doi:10.3390/diagnostics11020149)

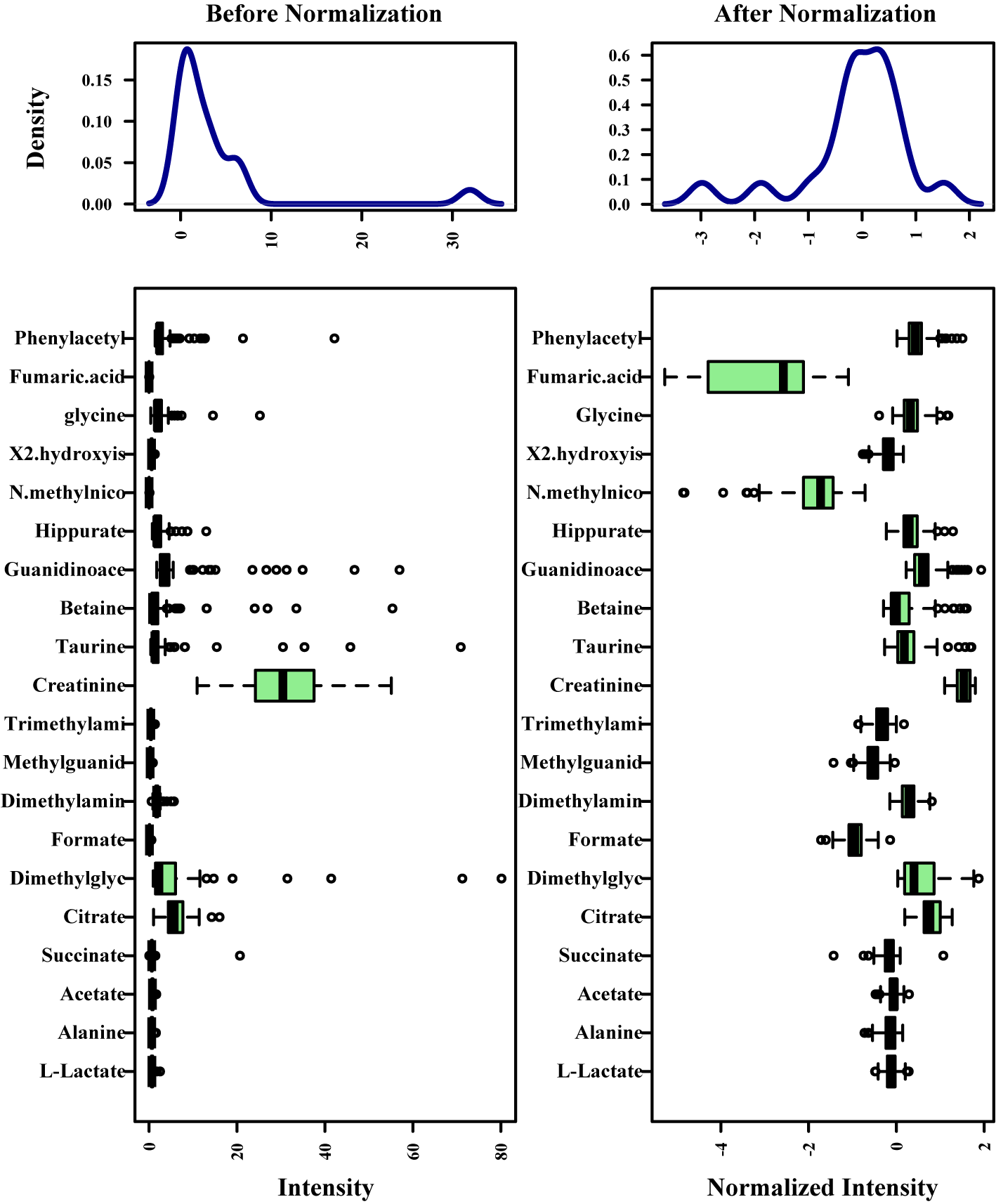


Figure S1: Data normalization. Density and intensity before and after data normalization

Supplement: Supplementary file 1 [file diagnostics-11-00149-s001.zip › Figure S1.docx]
